# Renal neuroendocrine control of desiccation and cold tolerance by Drosophila suzukii

**DOI:** 10.1002/ps.4663

**Published:** 2017-08-29

**Authors:** Selim Terhzaz, Lucy Alford, Joseph GC Yeoh, Richard Marley, Anthony J Dornan, Julian AT Dow, Shireen A Davies

**Affiliations:** ^1^ Institute of Molecular, Cell and Systems Biology, College of Medical, Veterinary and Life Sciences University of Glasgow Glasgow UK

**Keywords:** Drosophila, neuropeptides, Malpighian tubules, desiccation, cold tolerance

## Abstract

**BACKGROUND:**

Neuropeptides are central to the regulation of physiological and behavioural processes in insects, directly impacting cold and desiccation survival. However, little is known about the control mechanisms governing these responses in Drosophila suzukii. The close phylogenetic relationship of D. suzukii with Drosophila melanogaster allows, through genomic and functional studies, an insight into the mechanisms directing stress tolerance in D. suzukii.

**RESULTS:**

Capability (Capa), leucokinin (LK), diuretic hormone 44 (DH_44_) and DH_31_ neuropeptides demonstrated a high level of conservation between D. suzukii and D. melanogaster with respect to peptide sequences, neuronal expression, receptor localisation, and diuretic function in the Malpighian tubules. Despite D. suzukii's ability to populate cold environments, it proved sensitive to both cold and desiccation. Furthermore, in D. suzukii, Capa acts as a desiccation‐ and cold stress‐responsive gene, while DH_44_ gene expression is increased only after desiccation exposure, and the LK gene after nonlethal cold stress recovery.

**CONCLUSION:**

This study provides a comparative investigation into stress tolerance mediation by neuroendocrine signalling in two Drosophila species, providing evidence that similar signalling pathways control fluid secretion in the Malpighian tubules. Identifying processes governing specific environmental stresses affecting D. suzukii could lead to the development of targeted integrated management strategies to control insect pest populations. © 2017 The Authors. *Pest Management Science* published by John Wiley & Sons Ltd on behalf of Society of Chemical Industry.

## 1 INTRODUCTION

*Drosophila suzukii* (Matsumura), also known as the spotted wing *Drosophila*, is a serious pest of soft fruit worldwide.^1^ Although originally of Asian origin, the species has since become invasive in North America and continental Europe.^2^, ^3^, ^4^ *Drosophila suzukii* females inflict substantial crop damage by ovipositing eggs into ripe, pre‐harvest fruits via a specialised serrated ovipositor.^5^, ^6^ This damage is then exacerbated by the hatched larvae feeding on the fruit, resulting in substantial economic losses.^7^ Compared with *D. suzukii*, the physiological responses of *Drosophila melanogaster* to environmental stressors such as cold and desiccation have been well characterised.^8^ *Drosophila melanogaster*, which phylogenetically is very closely related to *D. suzukii*, is known to be chill susceptible and incapable of surviving internal ice formation.^9^ However, adult females can ‘overwinter’, that is, survive periods of sustained cold temperatures and associated desiccation, by entering a weak diapause, that is intermediate between a true photoperiodic diapause and a temperature‐driven quiescence, whereby they depress their metabolic rate, accumulate energy reserves, suppress ovarian development, and increase melanisation of the cuticle..^10^, ^11^ To survive the multiple‐stress environment of winter, *Drosophila* species exhibit enhanced stress tolerances and have many physiological and genetic mechanisms underlying cold and desiccation tolerance. Both cold and desiccation are closely linked at the molecular level to adjustments in the expression of genes involved in key physiological responses, for example in ion transport, carbohydrate metabolism, antioxidant production, immunity, signalling and gene expression pathways.^12^, ^13^, ^14^, ^15^, ^16^, ^17^ Recent work has identified neuroendocrine signalling as a control mechanism of environmental stress responses.^18^, ^19^, ^20^, ^21^

The ability to rapidly respond to, and tolerate over time, abiotic stresses such as temperature fluctuations (and associated alterations in food/water availability), in the short term and/or as a seasonal variation, requires insect osmoregulatory systems, including the gut and Malpighian (renal) tubule (MT), to act in a dynamic and appropriate manner to effect the physiological processes necessary to ensure the animals' survival. Insect osmoregulation, that is, the regulation of cellular ion and water homeostasis via transporters and water channels, is subject to highly sophisticated endocrine control.^22^ In *D. melanogaster*, several families of neuropeptides have been identified that regulate diuresis in the MTs, including the diuretic hormones DH_31_ 23 and DH_44_,^24^ the Capability (Capa)‐related peptides^25^ and the leucokinin (LK) neuropeptide.^26^ The recent transcriptomics and peptidomic analyses of the neuropeptides and their cognate receptors in *D. suzukii* have allowed identification of orthologous peptides and receptors, which in turn facilitates functional studies to identify suitable targets for the development of novel control strategies against this invasive pest.^27^

The depth of knowledge garnered from the genetic model organism *D. melanogaster* can readily be used to identify fundamental similarities that may exist in *D. suzukii*. Here, we describe the peptide sequences, morphology and organisation of the Capa, LK, and DH_44_‐producing neurons in the larval and adult central nervous system (CNS) of *D. suzukii* to gain better insight into the structure–function relationship of these neuropeptides. We also determined the MT‐specific cell types that receive Capa, LK, DH_44_ and DH_31_ neuropeptide signals and their physiological role in modulating tubule fluid homeostasis. Furthermore, we investigated tolerance to desiccation and cold in both *D. melanogaster* and *D. suzukii*, and we provide data suggesting that neuroendocrine responses to these stresses are potentially altered by the action of neuropeptides acting on the MT to regulate water and ion homeostasis critical for the animal's survival.

## 2 MATERIALS AND METHODS

### 2.1 Fly stocks

*Drosophila melanogaster* wild‐type Canton‐S (CS) flies were obtained from Bloomington Stock Center (Indiana University, Bloomington, USA) and maintained on a standard *Drosophila* medium at 22 °C (unless otherwise stated) and 45‐55% relative humidity with a 12:12 h light:dark photoperiod. *Drosophila suzukii* flies, originally collected in Italy, were obtained from the laboratory of R. Elwyn Isaac (University of Leeds, Leeds, UK) and reared on standard cornmeal agar medium supplemented with a blueberry fruit, at 26 °C and 60% humidity with a 12:12 h light:dark photoperiod. Adult *Drosophila* (1‐2 weeks post‐eclosion) of both sexes were used in this study.

### 2.2 Immunostaining

Immunostaining procedures were performed as described previously.^25^ Adult MTs and larval and adult nervous systems were dissected in Schneider's medium (Thermo Fisher Scientific, Paisley, UK) and fixed with 4% (w/v) paraformaldehyde (PAF) for 30 min at room temperature. Rhodamine‐labelled purified rabbit anti‐DH_44_ (1:2000^24^), rhodamine‐labelled purified rabbit anti‐Capa precursor peptide (dilution 1:500^25^), and rabbit anti‐LK (1:1000^26^) were used. Alexa Fluor 405/564‐conjugated affinity‐purified goat anti‐mouse and anti‐rabbit antibodies (Thermo Fisher Scientific) were used in a dilution of 1:1000 for visualisation of the primary antiserum. Incubations in the primary and secondary antibodies were performed overnight. Tubules were incubated with the nuclear stain 4',6‐diamidino‐2‐phenylindole (DAPI; 1 µg mL^−1^ for 1 min; Sigma‐Aldrich, Irvine, UK). Samples were mounted on poly‐l‐lysine (Sigma‐Aldrich)‐covered 35‐mm glass‐bottomed dishes (MatTek Corporation, MA, USA) in Vectashield (Vector Laboratories Inc, CA, USA). Confocal images were taken using an LSM 880 inverted microscope (Zeiss) and processed with Zen black/blue software (Zeiss, Oberkochen, Germany) and Adobe Photoshop/Illustrator CS 5.1.

### 2.3 Peptide synthesis and fluorescent‐tagged neuropeptide labelling

*Drosophila* Capa‐1, LK, DH_44_ and DH_31_ (both with and without N‐terminal cysteine) were synthesised by Cambridge Peptides (Birmingham, UK). The amino acid sequences used to synthesise the neuropeptides were based on the *D. melanogaster* sequences as most of the peptides predicted and identified from *D. suzukii* appear to be identical to those previously characterised from *D. melanogaster*.^27^ The protocol and generation of neuropeptide conjugated to a high quantum yield fluorophore have been described previously.^28^ The fluorescent‐tagged neuropeptides generated were TMR‐C_5_‐maleimide‐Capa‐1, Alexa‐488‐C5‐maleimide‐LK, TMR‐C_5_‐maleimide‐DH_44_ and TMR‐C_5_‐maleimide‐DH_31_. Ligand receptor binding assays were performed on live MTs dissected from 6 − 7‐day‐old adult females. Tubules were mounted on poly‐L‐lysine coated glass‐bottomed dishes in phosphate‐buffered saline (PBS) and then incubated for 2 minutes in a 1:1 mixture of Schneider's medium:*Drosophila* saline containing 500 ng/ml DAPI and 10^‐7^ m fluorescent‐tagged neuropeptide and subsequently with 10^−5^ m unlabelled neuropeptides. As the staining is transitory, the samples were imaged immediately after treatment. All images were taken using a Zeiss LSM 810 inverted confocal microscope.

### 2.4 Fluid secretion assay

Secretion assays were performed as described previously.^29^ MTs from 6 − 7‐day‐old adult female *D. suzukii* flies were dissected under Schneider's medium and isolated into 10‐µl drops of a 1:1 mixture of Schneider's medium:*Drosophila* saline. Intact tubules were left to secrete for approximately 30 min, with non‐secreting tubules being replaced if necessary, to produce a set of 10 − 15 working tubules. Secretion rates were measured every 10 min, then the diuretic *Drosophila* Capa‐1, LK, DH_44_ and DH_31_ neuropeptides were added to 10^−7^ m, and secretion rates were measured for a further 30 min. Data are plotted as mean ± standard error of the mean (SEM).

### 2.5 Gravimetric method of estimating body water

To measure wet body weight, *D. melanogaster* and *D. suzuki* 6‐day‐old normally fed female flies were anaesthetised and weighed on an AND GR‐202 (Oxford, UK) precision balance (analytical weighing to within 0.0001 g). Another group of flies of each *Drosophila* species were weighed after 18 h of desiccation with no food and no water. Water loss over 18 h was calculated for each group by subtracting the water content at 18 h from that at 0 h. Experiments were run in triplicate with at least 10 flies of each *Drosophila* species.

### 2.6 Assays of gene expression for cold and desiccation stress

#### 2.6.1 *Cold*

*Drosophila suzukii* 6‐day‐old female flies were untreated (control), chilled at 4 °C for 6 h (cold), or chilled at 4 °C for 6 h and subsequently allowed to recover for 24 h (recovery). Before treatment, both *D. suzukii and D. melanogaster* were maintained at a culture temperature of 26 °C to enable a direct species comparison and avoid any potential effects of rearing temperature on inherent thermal tolerance. No mortality was observed for the different treatment groups.

#### 2.6.2 *Desiccation*

*Drosophila suzukii* 6‐day‐old female flies were either untreated (control), placed in empty vials (no food or water) for 18 h at 21 °C and 45‐55% relative humidity (desiccation), or exposed to desiccation for 18 h and subsequently allowed to recover for 24 h (recovery). Before treatment, both *D. suzukii and D. melanogaster* were maintained at a culture temperature of 26 °C to enable a direct species comparison and avoid any potential effects of rearing temperature and relative humidity on inherent thermal and desiccation resistance. Following treatment, groups of 10 flies were transferred immediately to microcentrifuge tubes containing TRIzol Reagent (Thermo Fisher Scientific) for RNA extraction. For *D. suzukii* whole‐fly mRNA expression, quantitative reverse transcriptase − polymerase chain reaction (qRT‐PCR) amplifications were performed in an ABI StepOnePlus Detection System (Applied Biosystems, CA, USA) with Brilliant III Ultra‐Fast SYBR Green QPCR master mix (Agilent, Edinburgh, UK) using the following primer pairs: *Capa*, 5'‐CCGAGTCTGGCAAACAGTCTG‐3' and 5'‐ GACACCAACAAGGGACAAAAGG‐3'; *LK*, 5'‐CAGTTTCTGGACAGGATTCGGAG‐3' and 5'‐ CAGGTCTCGCATTGTAGGCATC‐3; *DH*_*31*_, 5'‐ATGCCCAGCCAATCCAATGGTG‐3' and 5'‐ TGCGTCCAAAGCGAGTCATC‐3'; *DH*_*44*_ *,* 5'‐CTTCTGCTGGAAATCGCACG‐3' and 5'‐ GTTGCTCCAAACCCTCCA‐3'; Ribosomal protein L18 (*rpl18*), 5'‐GTTGCTCCAAACCCTCCA‐3' and 5'‐ GATCCGTCTAACACCTCCC ‐3'. Data were normalised against the *D. suzukii rpl18* standard and expressed as fold change compared with controls ± SEM (*n* = 3).

### 2.7 Survival under different stressors

#### 2.7.1 *Cold*

Cold tolerance was determined via calculation of the lower lethal temperature (LLT_50_), i.e. the low temperature that results in 50% mortality of a test population. Adults of *D. suzukii* and *D. melanogaster*, both reared at 26 °C, were selected at 6 days post‐eclosion, respectively, and exposed to a range of low temperatures (‐10 °C to ‐2 °C at 1 °C intervals) using a direct plunge method.^30^, ^31^ Temperature ranges were selected to encompass 0 − 100% mortality. For each temperature treatment, 30 adults of each sex and each species were anaesthetised briefly with CO_2_ and placed in Eppendorf tubes at densities of ten adults per tube, which, in turn, were placed within a glass boiling tube held within an alcohol bath (Haake G50 and PC200; Thermo Scientific, Karlsruhe, Germany) pre‐set to the desired temperature. Adults were held at the desired exposure temperature for 1 h. Following exposure, adults were allowed to recover in culture vials containing a food source and survival was assessed after 48 h. The procedure was repeated for each exposure temperature.

#### 2.7.2 *Desiccation*

*Drosophila suzukii* and *D. melanogaster* 6‐day‐old male and female flies were anaesthetised briefly with CO_2_ and placed in groups of 30‐40 in 30‐mL empty vials (no food or water), and the open end of the tube was sealed with parafilm (Bemis, NA, USA). Vials were checked hourly until no living flies remained. All experiments were run in triplicate with at least 30 flies in each run for each *Drosophila* species.

### 2.8 Bioinformatics

Neuropeptide precursor sequences were obtained from DINeR (http://www.neurostresspep.eu/diner) and/or from the National Center for Biotechnology Information (NCBI).^32^ Sequences were aligned using the multiple sequence alignment program Clustal Omega. 33

### 2.9 Statistical analysis

For fluid secretion analysis and for the water retention assay, a two‐tailed Student's *t*‐test, taking *P* = 0.05 as the critical value (for two independent groups: basal versus stimulated), was used. For mRNA level quantification, one‐way analysis of variance (ANOVA) followed by Tukey's multiple comparisons of means with a significance level of *P* < 0.05 (for three independent groups: nonstressed versus stressed versus recovered) was used to compare neuropeptide mRNA levels in cold or desiccated flies. For survival curves obtained in desiccation assays, significance was assessed by the log‐rank (Mantel–Cox) test. Log‐rank tests were conducted for each pairwise comparison. The statistical tests were performed using GraphPad Prism 7.0 software (GraphPad Software Inc., CA, USA). For cold shock experiments, the temperature resulting in 50% mortality of experimental populations (LLT_50_) was determined for low exposure temperatures using Probit analysis in MINITAB, version 17 (Minitab Inc., State College, PA, USA). Handling controls resulted in approximately 99% survival across all treatments. The natural response rate was therefore assumed to be close to zero and was not included in the model. Significant differences in mortality were identified by non‐overlapping 95% fiducial limits.

## 3 RESULTS

### 3.1 Neuropeptide precursor data

**Figure 1:**
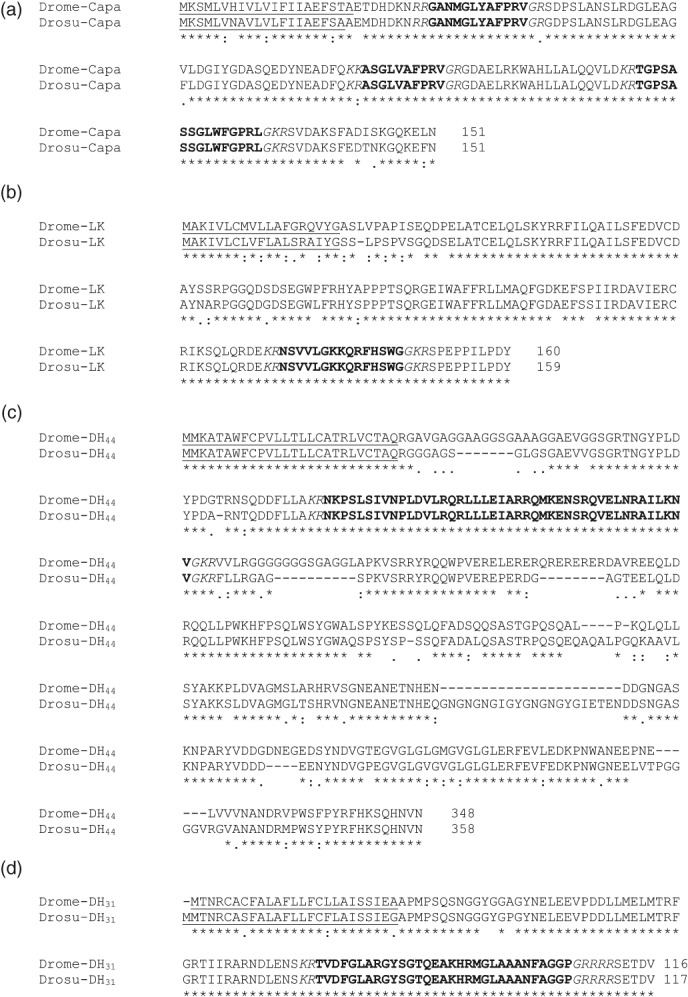
Alignment of amino acid sequences encoded by D. suzukii (Drosu) and D. melanogaster (Drome) neuropeptide precursors identified from NCBI for (a) Capa, (b) leucokinin (LK), (c) DH_44_, and (d) DH_31_ prepropeptides. Predicted peptide sequences are highlighted in bold, potential endoproteolytic cleavage sites are in italics, and the signal peptide sequence is underlined. GenBank Accession numbers: Drome‐Capa (AAF56969.2), Drosu‐Capa (XP_016937441.1), Drome‐LK (AAF49731.2), Drosu‐LK (XP_016933307.1), Drome‐DH_44_ (NP_649922.2), Drosu‐DH_44_ (XP_016930975.1), Drome‐DH_31_ (Q9VLK4.1) and Drosu‐DH_31_ (XP_016944990.1).

Amino acid BLAST searches of the *D. suzukii* protein databases revealed the precursor sequences of Capa,^34^ LK, DH_44_ and DH_31_ neuropeptide families. A comparison of *D. suzukii* and *D. melanogaster* Capa, LK, DH_44_ and DH_31_ peptide precursors is shown in Fig. 1. We found that the amino acid sequences of the *D. suzukii* predicted peptides are identical to the sequences found in *D. melanogaster*. Furthermore, the presence of these peptides and/or peptides precursors in *D. suzukii* has previously been confirmed by mass spectrometric analysis. 27

### 3.2 Capa, LK and DH_44_ neuropeptide‐expressing neurons

To gain insight into the physiological function of Capa, LK and DH_44_ neuropeptides in *D. suzukii*, we determined the localisation of the neurons producing these neuropeptides and their projections. As the peptide precursor sequences are very similar between *D. suzukii* and *D. melanogaster*, we used available antisera against *D. melanogaster* Capa, LK, and DH_44_ on the *D. suzukii* larval and adult CNS.

**Figure 2:**
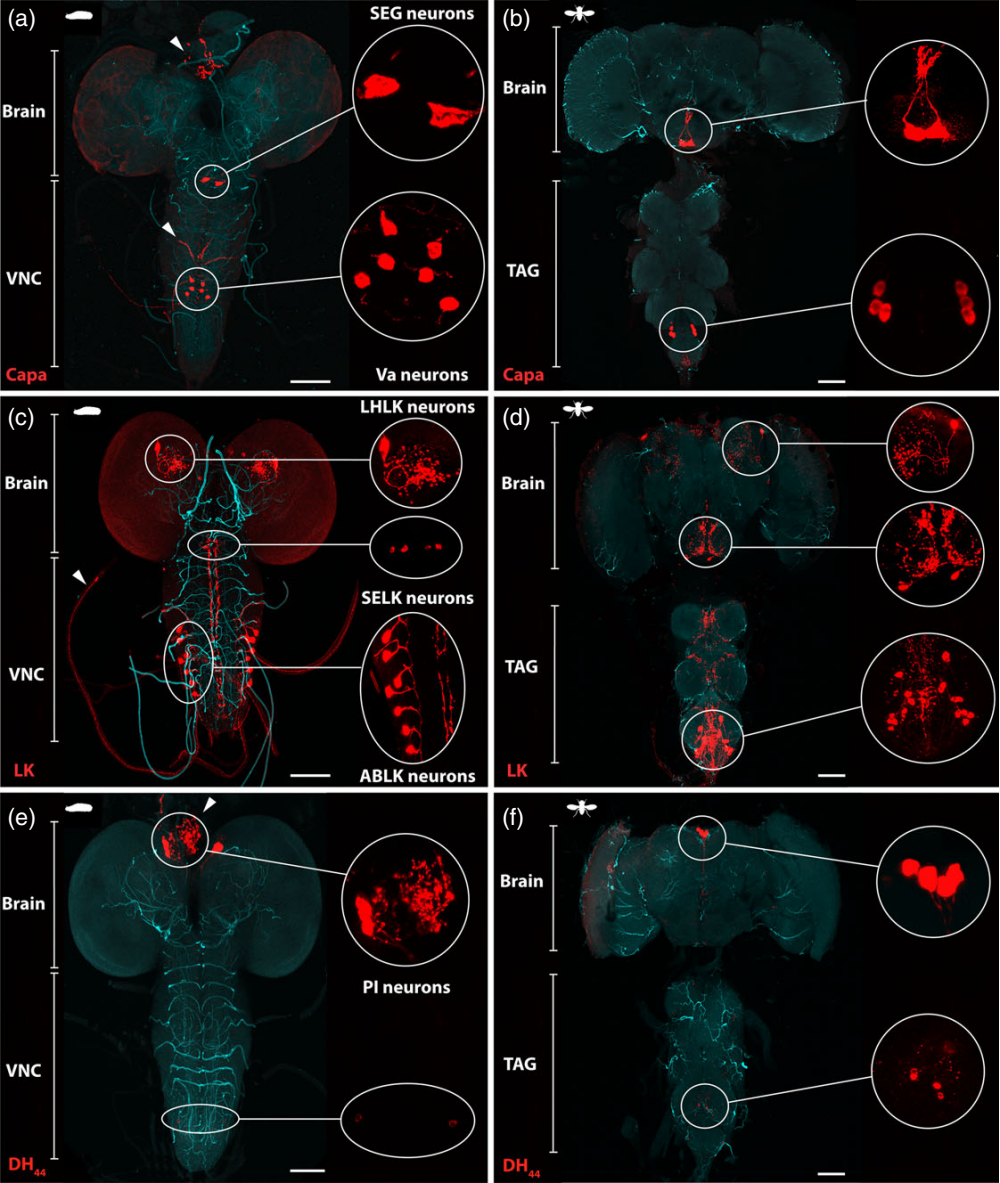
Immunocytochemical localisation of Capa, LK and DH_44_ neuropeptides in the D. suzukii larval and adult central nervous system (CNS). (a, b) Localisation of Capa neurons using anti‐Capa precursor antibody in (a) the larval and (b) the adult CNS. Capa precursor immunoreactivity is localised to a single pair of very large neuroendocrine cells in the suboesophageal ganglion (SEG neurons) and three pairs of abdominal neuroendocrine cells (Va neurons). Arrowheads show neurohaemal organs, the retrocerebral complex for the two cell bodies in the suboesophageal neuromere, and the abdominal median nerves for the three pairs of ventral neuroendocrine cells in the abdominal neuromeres. (c, d) LK immunofluorescence using anti‐LK antibody in (c) the larval and (d) the adult CNS. LK‐expressing neurons are distributed as a pair of lateral horn LK (LHLK) neurons, two pairs of suboesophageal LK (SELK) neurons, and seven pairs in the abdominal ganglia of the larval CNS. In the ventral ganglion of the adult, around nine pairs of abdominal LK (ABLK) neurons have axons that leave the CNS by abdominal nerves and processes for the release of LK into the haemolymph. (e, f) Localisation of DH_44_ neurons using anti‐DH_44_ antibody in (e) the larval and (f) the adult CNS. DH_44_ peptides are produced in six neurons (three cells in each cerebral lobe) of the pars intercerebralis (PI), with axons running to the retrocerebral complex of the corpus cardiacum (arrow). Additional smaller set of neuroendocrine cells in the larval and adult ventral nerve cord are labelled with the DH_44_ antibody. All patterns of expression are representative of both males and females. Inserts are maximum projections of confocal z‐series showing magnifications of selected regions. VNC, ventral nerve cord; TAG, thoracic‐abdominal ganglion. Scale bars = 100 µm.

The antiserum to the Capa precursor recognised several neurons in the larval and adult CNS (Fig. 2a and b), consisting of three pairs of ventral neuroendocrine cells in the abdominal neuromeres (Va neurons), and a single pair of large neuroendocrine cells in the suboesophageal ganglion (SEG neurons). In the larva, the Va neurons use the surface of the three pairs of abdominal median nerves as their neurohaemal release site (Fig. 2a, arrowhead), while in the adult, the dorsal surface of the thoracicoabdominal ganglion is used. In larvae and adults, the axons of the SEG neurons project to the cerebral ganglion and project from the brain by means of the nervi corporis cardiac to the retrocerebral complex (Fig. 2a, arrowhead), which allows the release of the Capa neuropeptides into the haemolymph.

In this study, LK‐immunoreactive cells were identified using an antibody prepared against leucokinin I, which shows broad specificity for the LK family of neuropeptides. ^26,^ 35 LK expression was observed in the brain and ventral ganglion of the *D. suzukii* CNS (Fig. 2c and d). One pair of large protocerebrum neurons located in the lateral horn leucokinin (LHLK) area surrounds the peduncles of the mushroom bodies and two pairs of suboesophageal leucokinin (SELK) neurons project extended processes to the tritocerebrum and through the cervical connection to the ventral ganglion. In the larva, the ventral ganglion showed one pair of prominent LK‐immunoreactive neurons in seven abdominal leucokinin (ABLK) neuromeres that send their processes to the lateral abdominal nerves (Fig. 2c, arrowhead). In the ventral ganglion of the adult, around nine pairs of abdominal neurons have axons that project from the CNS via the abdominal nerves and processes that connect to each other, both ipsi‐ and contra‐laterally. The latter neurons appear to be primarily responsible for the release of LK into the haemolymph.

Using a specific anti‐DH_44_ antibody, DH_44_ expression was observed in a restricted number of neurons within the larval and adult CNS (Fig. 2e and f), most notably in a bilateral triplet of neurons localised to the pars intercerebralis (PI) and the neurohaemal axons and axon terminals in the retrocerebral complex (Fig. 2e, arrowhead). In addition, while no DH_44_ immunoreactive cells were found in the abdominal ganglia by *in situ* hybridisation, we found additional pairs of small neurons in the larval and adult ventral ganglion (Fig. 2e and f, higher magnification of merge confocal stacks), similar to those found in *D. melanogaster* CNS expressing membrane‐bound green fluorescent protein (GFP) driven by DH_44_‐GAL4.^21^ Interestingly, the six DH_44_ neurons of the PI also express the LK receptor.^24^

### 3.3 Receptor mapping assay using fluorescently labelled neuropeptides

**Figure 3:**
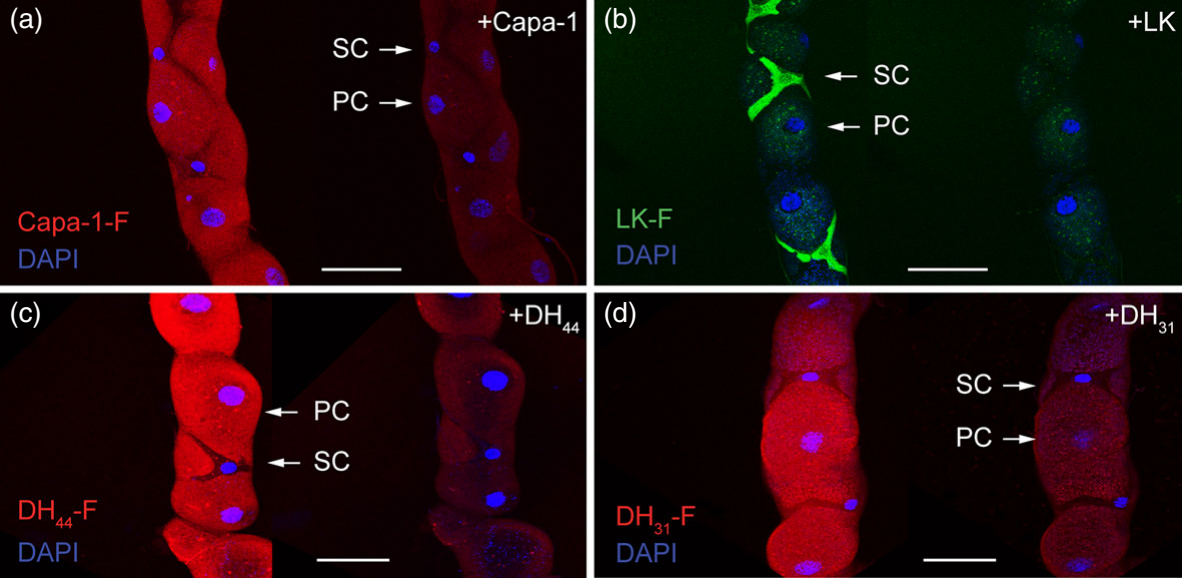
Fluorophore‐labelled Drosophila neuropeptides show receptor localisation in renal (Malpighian) tubules of D. suzukii. A receptor mapping assay using Capa‐1‐F, DH_44_‐F, and DH_31_‐F fluorescently tagged neuropeptides (10^−7^ m; red) confirmed that the labelled neuropeptides bind to principal cell (PC) basolateral membranes, while the stellate cells (SCs) are specifically labelled with 10^−7^ m of LK‐F (green). Excess unlabelled (a) Capa‐1, (b) leucokinin (LK), (c) DH_44_, and (d) DH_31_ neuropeptides (10^−5^ m) displace fluorescent signal in a ligand competition assay, thus confirming the specificity of binding. DAPI was used for nuclear staining (blue). Scale bars = 30 µm.

It has previously been demonstrated in *D. melanogaster* that Capa, LK, DH_44_ and DH_31_ neuropeptides are released into the circulation from neurohaemal release sites, whereupon they bind their cognate G protein‐coupled receptors localised at the MTs.^36^ The MTs are characterised by two main physiologically distinct secretory cell types, the principal (PC) and secondary (stellate; SC) cells, which receive multiple separate neuropeptide signals.^28^ Employing a fluorescent ligand‐receptor binding assay to determine the neuropeptide signalling associated with these two cell types in *D. suzukii* MTs, we were able to demonstrate specificity of binding of Capa‐1 (one of the peptides encoded by the *Capa* gene), LK, DH_44_ and DH_31_ neuropeptides to tubule PCs and SCs. As in *D. melanogaster*, application of Capa‐1‐F, DH_44_‐F and DH_31_‐F maps their receptors to the basolateral membrane of PCs (Fig. 3a, c and d), whereas LK‐F specifically labels SCs (Fig. 3b). Specificity of the fluorescently tagged neuropeptide binding was verified by a ligand competition assay in which the respective unlabelled peptide was able to almost fully abolish the fluorescent signal (Fig. 3). These data are consistent with the known action of Capa‐1, DH_44_ and DH_31_ neuropeptides specific to the PCs, with LK confined to SCs, in *Drosophila* MTs where they act through independent, although complimentary, cell signalling pathways to increase primary urine production.^37^

### 3.4 **Effect of Capa, LK, DH**_**44**_ and **DH**_**31**_ **neuropeptides on fluid secretion**

**Figure 4:**
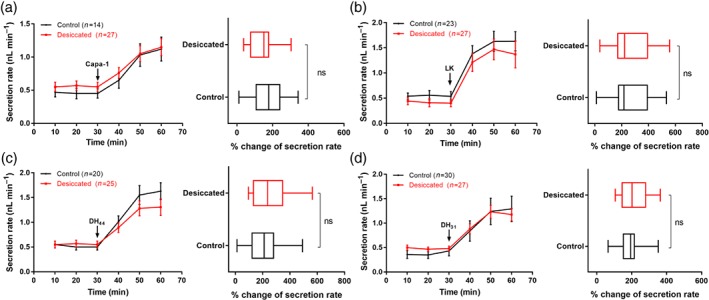
Effects of Drosophila neuropeptides on rates of fluid secretion by D. suzukii Malpighian tubules. Data showed the response of the tubule fluid secretion rate to (a) Capa‐1, (b) leucokinin (LK), (c) DH_44_, and (d) DH_31_, added to a final concentration of 10^‐7^ m at 30 min. All neuropeptides significantly increase the fluid secretion rate when applied to excised Malpighian tubules. Data are presented as mean ± SEM; P < 0.05. The percentage change in secretion rate following stimulation with 10^‐7^ m neuropeptides is similar in desiccated D. suzukii and non‐desiccated flies [non‐significant (ns)].

*Drosophila melanogaster* Capa‐1, LK, DH_44_ and DH_31_ neuropeptides were tested on their ability to stimulate fluid secretion on *D. suzukii* MTs. As expected, all four neuropeptides significantly increased the fluid secretion rate over 30 min (Fig. 4 a − d), with the extent of these increases comparable to those observed in *D. melanogaster* tubules. Interestingly, the greatest stimulation of fluid secretion was induced by LK, a neuropeptide that modulates the ‘chloride shunt’ pathway specifically in *D. melanogaster* SCs, resulting in a rapid collapse of the transepithelial potential and a concomitant increase in fluid secretion.^38^, ^39^ DH_44_, DH_31_ and the Capa‐1 neuropeptides act on their cognate G‐protein coupled receptor localised in the PCs. Activation of the PCs alone produces only a modest increase in fluid secretion, as the resting chloride conductance remains relatively low.

The impact of desiccation on MT function was also assessed via secretion assays. The baseline and neuropeptide‐stimulated secretion rate of *D. suzukii*, exposed to 18 h of desiccation, was not significantly lower than that of unstressed flies. Indeed, the percentage change in secretion rates following stimulation with the different neuropeptides was similar in desiccated and non‐desiccated flies (Fig. 4). Intriguingly, while neuropeptides significantly increase the fluid secretion rate to a similar extent when applied to MTs of the two species exposed to desiccation,^21^, ^28^ the baseline secretion rates are significantly lower in *D. melanogaster* flies; this suppression of basal fluid secretion is not apparent in *D. suzukii*. Hence, the direct action of these neuropeptides to *D. suzukii* MTs suggests that excretory water loss may contribute significantly to desiccation resistance.

### 3.5 Desiccation and cold tolerance

**Figure 5:**
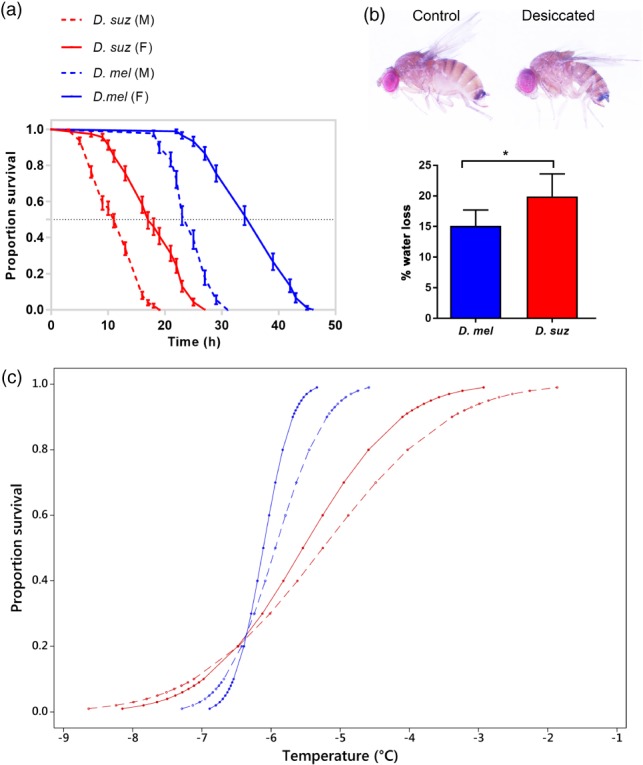
Desiccation and cold tolerance in D. suzukii and D. melanogaster. (a) Desiccation resistance was significantly higher in D. melanogaster compared with D. suzukii and in females compared with males for both species (P < 0.0001; log‐rank test; n = 90 − 130 flies for each Drosophila species). (b) Phenotype of D. suzukii desiccated for 18 h and water loss for D. melanogaster and D. suzukii exposed to desiccation. A significant increase in water loss was seen in D. suzukii flies; *P < 0.05. (c) Survival curves for D. melanogaster and D. suzukii. Cold tolerance was significantly higher in D. melanogaster compared with D. suzukii, while no sex effect was apparent. Drosophila melanogaster is indicated by the blue lines and D. suzukii by the red lines. For each species, females are indicated by the block line and males by the dashed line.

As Capa and kinin have been previously shown to modulate stress tolerance in *D. melanogaster*, we investigated comparative survival following desiccation and cold stress between *D. melanogaster* and *D. suzukii* male and female flies. During desiccation stress, *D. melanogaster* survived longer than *D. suzukii*, with a significant sexually dimorphic effect observed for both *Drosophila* species, with females exhibiting enhanced desiccation resistance (Fig. 5a; median survival time: *D. melanogaster* females, 39 h; *D. melanogaster* males, 25 h; *D. suzukii* females, 17 h; *D. suzukii* males, 11 h). In desiccating environments, a key mechanism used by insects to maintain water balance is to reduce the rate of water loss.^40^ We then performed water loss measurements in flies desiccated for 18 h compared with normally fed female flies for both *Drosophila* species (Fig. 5b; body weight of individual adult female fly (mean ± SEM): *D. melanogaster*, 1.02 ± 0.05 mg; *D. melanogaster* desiccated, 0.86 ± 0.04 mg; *D. suzukii*, 1.89 ± 0.16 mg; *D. suzukii* desiccated, 1.51 ± 0.13 mg). During desiccation stress, water loss in *D. suzukii* was significantly higher than in *D. melanogaster* (20% and 15%, respectively, compared with nonstressed control groups; *P* < 0.05). During cold exposure, *D. melanogaster* proved significantly more cold tolerant than *D. suzukii*, as determined by non‐overlapping 95% fiducial limits (Fig. 5c). No significant difference between the cold tolerance of males and that of females was observed for either species (LLT_50_: *D. melanogaster* females, ‐6.1 °C; *D. melanogaster* males, ‐5.9 °C; *D. suzukii* females, ‐5.5 °C; *D. suzukii* males, ‐5.2 °C). For *D. melanogaster*, 5 − 95% mortality occurred over a temperature range of 1.1 °C for females and 1.9 °C for males. For *D. suzukii*, the corresponding mortality level occurred over a temperature range of 3.7 and 4.8 °C, respectively.

### 3.6 Effect of desiccation and cold stress on neuropeptide mRNA levels

**Figure 6:**
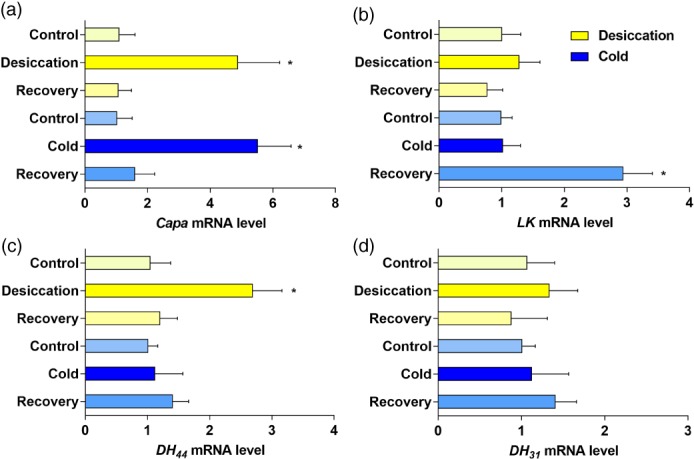
Effect of desiccation and cold stress on D. suzukii neuropeptide mRNA levels. Quantitative RT‐PCR analysis of RNA extracted from whole flies exposed to 18 h of desiccation, to cold shock of 4 °C for 6 h, to desiccation/cold stress with subsequent recovery for 24 h, or to no treatment for (a) Capa‐1, (b) leucokinin (LK), (c) DH_44_, and (d) DH_31_ was performed. Following desiccation, Capa and DH_44_ mRNA expression was significantly increased with no impact on LK and DH_31_ expression. Following cold exposure, Capa mRNA levels were up‐regulated while LK, DH_44_ and DH_31_ expression mRNA levels were unchanged. Interestingly, LK mRNA levels were up‐regulated during recovery from a cold challenge. Data are expressed as mean fold change compared with non‐stressed controls ± SEM (n = 3). Asterisks indicate a significant increase (*P < 0.05; Student's t‐test) compared with the non‐stressed control.

MTs are critical organs for epithelial fluid transport and stress tolerance in insects, and recent studies have described the mechanism in renal tubule epithelia that enhances both desiccation and cold survival.^20^, ^41^ Given that tubule function is controlled by multiple neuropeptides, putative roles for Capa, LK, DH_44_ and DH_31_ signalling in desiccation and cold stress were explored (Fig. 6) by measuring their mRNA expression levels, after exposure to 18 h of desiccation at 21 °C, or after exposure to a cold shock of 4 °C for 6 h, or after exposure to desiccation/cold stress and subsequent recovery for 24 h. We found that *Capa* and *DH*_*44*_ mRNA expression increased significantly following desiccation stress (4.8‐ and 2.7‐fold, respectively, compared with the nonstressed control groups; *P* < 0.0001), while *LK* and *DH*_*31*_ expression remained unchanged. Following cold exposure, *Capa* mRNA levels were up‐regulated (> 5‐fold compared with untreated controls; *P* < 0.0001), while cold exposure had no significant effect on *LK*, *DH*_*44*_ or *DH*_*31*_ expression. Surprisingly, *LK* mRNA levels were up‐regulated during recovery after cold challenge (Fig. 6b).

## 4 DISCUSSION

Research into the fruit pest *D*. *suzukii* has intensified in line with the continued global expansion of this species and the associated increased economic costs incurred as a consequence of crop damage. Much of this interest has focussed on *D. suzukii's* ability to tolerate climatic conditions specific to the new geographical areas it is invading, focussing particularly on overwintering behaviours and the species' responses to cold and desiccation environmental stresses.^42^, ^43^, ^44^ Here, by comparing the thermal stress tolerance of our Canton‐S *D. melanogaster* with that of *D. suzukii*, we show that *D. melanogaster* seems to survive at significantly lower temperatures than *D. suzukii*. However, these results were solely obtained from a single strain of each *Drosophila* species and one should be cautious about making species‐level conclusions because of the considerable genetic variation in stress traits across different populations of *D. melanogaster* and *D. suzukii* and even between different genotypes obtained from the same population.^45^, ^46^ Nevertheless, females of *D. suzukii* have an LLT_50_ of ‐5.5 °C, and therefore were capable, at least for a short time, of surviving temperatures below those previously observed, where survival was shown to be limited below temperatures of 10 °C. 47, 48

It should be noted that experiments were performed on individuals of both species maintained at temperatures >20 °C, corresponding to summer conditions. Recent research has shown that *D. suzukii* is capable of producing a winter morph phenotype when the summer morph is reared at temperatures of ≤10 °C, and that such a phenotype may aid winter survival and explain why *D. suzukii* has proved successful in cold climates.^42^, ^43^ However, knowledge of the thermotolerance and thermal physiology of this winter phenotype is currently limited. A recent study has revealed the winter phenotype to display enhanced cold tolerance when compared with the summer phenotype with regard to measurement of the LLT_50_, although reduced cold tolerance with regard to measurement of the supercooling point (the temperature of crystallisation) and with substantial mortality occurring above the supercooling point.^42^ The authors suggest that the winter phenotype may not invest in cryoprotectants to prevent freezing, but instead may utilise mechanisms to survive chill injury. How the cold tolerances of *D. suzukii* and *D. melanogaster* differ when in their respective winter phenotypes is presently unknown. However, at least in their summer morphs, our lab‐reared Canton‐S *D. melanogaster* is significantly more cold tolerant than *D. suzukii*.

Previous studies have presented contradictory descriptions comparing levels of cold tolerance between the sexes in *D. melanogaster*, with some studies observing that males demonstrate enhanced cold tolerance^49^, ^50^ and others the converse.^51^, ^52^ To clarify this, we tested male and female populations for both *D. melanogaster* and *D. suzukii* and observed no significant difference in cold tolerance between the sexes for both species. This apparent discrepancy may be a consequence of variation in the indices applied to the individual cold tolerance assays, i.e. chill coma, supercooling point, lethal time, lethal temperature etc.,^53^, ^54^, ^55^, ^56^ and/or variance in experimental factors, e.g. the age of the flies or the temperature at which they are tested.^50^, ^57^

In keeping with our observation that *D. melanogaster* proved hardier in response to cold stress, we also observed that *D. melanogaster* was significantly more tolerant to desiccation than *D. suzukii*. Indeed, *D. suzukii* proved highly sensitive to desiccation, with complete lethality occurring in both sexes after approximately 24 h of desiccation (as opposed to *D. melanogaster* which survived approximately twice as long). Our data suggest that this increased sensitivity to desiccation is a consequence of the observed higher level of fluid loss in *D. suzukii* as compared with *D. melanogaster*. It has been shown that, under desiccation conditions, diuretic neuropeptide receptor expression in *D. melanogaster* tubules is decreased, with a concomitant reduction of the basal tubule fluid secretion rate, therefore potentially limiting water loss.^20^, ^21^ The lack of any physiological response to lower basal secretion rates in *D. suzukii* is surprising, and, although the reduced rate of water loss in desiccated *Drosophila* may be caused by combined reduction in respiratory, cuticular, and excretory water loss, it is possible that, given the unchanged *D. suzukii* basal secretion during desiccation stress, the MTs may play an important role in water balance during desiccation resistance.

Interestingly, whilst no difference was apparent in the cold tolerance responses of males and females, a significant sexually dimorphic effect was observed in *D. suzukii* during desiccation, with females exhibiting enhanced desiccation resistance, surviving for 8 h longer than males. This is supported by previous work which found female *D. suzukii* to be significantly more desiccation‐resistant. 58, 59 In insects, where sexual dimorphism occurs, males and females are known to differ in their desiccation resistance, with larger body size associated with enhanced desiccation resistance. 60 However, this does not hold true for starvation tolerance, as *D. melanogaster* males survived starvation longer than females, despite the smaller size of males.^61^

Diuretic neuropeptides that act on *D. melanogaster* MTs to modulate fluid homeostasis have also been found to modulate stress tolerance. Roles for Capa, LK and DH_44_ neuropeptide signalling in cold and desiccation has been recently demonstrated in *D. melanogaster*. Our data for *D. suzukii* indicate the involvement of Capa and DH_44_ signalling in desiccation, while Capa signalling is also involved in response to cold stress, as the expression levels for both neuropeptides were increased in response to these stresses and returned to basal levels within the recovery period. Surprisingly, *LK* mRNA levels were up‐regulated during the recovery period following a cold challenge, suggesting that LK neurons may release LK peptide during recovery from cold stress. As LK does have demonstrated roles in feeding behaviour^62^ and starvation tolerance^21^ and starvation can be an associated factor during winter survival, a potential novel role for LK in cold tolerance responses in *D. suzukii* would be a useful subject for future investigations. 63

In *Drosophila*, several peptide hormones have been identified as pivotal to regulating ion transport and fluid secretion in the MTs and gut.^29^, ^37^ Here, we describe in the pest species *D. suzukii* the neuropeptide sequences for Capa, LK, DH_44_ and DH_31_, the neuroendocrine cells that produce these neuropeptides, their processes and the potential neural network that affects water homeostasis (and also feeding behaviour), showing it to be similar to that of *D. melanogaster*. To fully determine the importance of these neuropeptides and receptor genes in *D*. *suzukii* biology, it will be necessary to develop functional genomic tools that are currently employed in *D*. *melanogaster*. Germline transformation techniques and Clustered Regularly Interspaced Short Palindromic Repeats/CRISPR‐associated proteins (CRISPR/Cas9)‐mediated mutagenesis have already been successfully deployed in *D*. *suzukii*,^64^ and this ability to manipulate gene expression using a reverse genetic approach has already proved to be a powerful tool for understanding gene function.^65^ Unravelling the molecular, physiological and behavioural processes controlled by neuropeptides during environmental stress may augment the development of potential routes for targeted pest control.
